# The Molecular Mechanisms of the Relationship between Insulin Resistance and Parkinson’s Disease Pathogenesis

**DOI:** 10.3390/nu15163585

**Published:** 2023-08-15

**Authors:** Viviana A. Ruiz-Pozo, Rafael Tamayo-Trujillo, Santiago Cadena-Ullauri, Evelyn Frias-Toral, Patricia Guevara-Ramírez, Elius Paz-Cruz, Sebastián Chapela, Martha Montalván, Tania Morales-López, Daniel Simancas-Racines, Ana Karina Zambrano

**Affiliations:** 1 Centro de Investigación Genética y Genómica, Facultad de Ciencias de la Salud Eugenio Espejo, Universidad UTE, Quito 170527, Ecuador; 2 School of Medicine, Universidad Católica Santiago de Guayaquil, Guayaquil 090615, Ecuador; 3 Departamento de Bioquímica, Facultad de Ciencias Médicas, Universidad de Buenos Aires, Ciudad Autónoma de Buenos Aires C1121ABE, Argentina; 4 Equipo de Soporte Nutricional, Hospital Británico de Buenos Aires, Ciudad Autónoma de Buenos Aires C1280AEB, Argentina; 5 School of Medicine, Universidad Espíritu Santo, Samborondón 091952, Ecuador; 6 Facultad de Ciencias de la Salud Eugenio Espejo, Universidad UTE, Quito 170527, Ecuador; 7 Centro de Investigación de Salud Pública y Epidemiología Clínica (CISPEC), Universidad UTE, Quito 170527, Ecuador

**Keywords:** Parkinson’s disease, insulin resistance, dopaminergic neurons, insulin signaling

## Abstract

Parkinson’s disease (PD) is a degenerative condition resulting from the loss of dopaminergic neurons. This neuronal loss leads to motor and non-motor neurological symptoms. Most PD cases are idiopathic, and no cure is available. Recently, it has been proposed that insulin resistance (IR) could be a central factor in PD development. IR has been associated with PD neuropathological features like α-synuclein aggregation, dopaminergic neuronal loss, neuroinflammation, mitochondrial dysfunction, and autophagy. These features are related to impaired neurological metabolism, neuronal death, and the aggravation of PD symptoms. Moreover, pharmacological options that involve insulin signaling improvement and dopaminergic and non-dopaminergic strategies have been under development. These drugs could prevent the metabolic pathways involved in neuronal damage. All these approaches could improve PD outcomes. Also, new biomarker identification may allow for an earlier PD diagnosis in high-risk individuals. This review describes the main pathways implicated in PD development involving IR. Also, it presents several therapeutic options that are directed at insulin signaling improvement and could be used in PD treatment. The understanding of IR molecular mechanisms involved in neurodegenerative development could enhance PD therapeutic options and diagnosis.

## 1. Introduction

The role of insulin resistance (IR) in Parkinson’s disease (PD) pathogenesis is unclear. This relationship has been previously reviewed [1,2,3,4,5,6,7,8]. The current review integrates and amplifies the eight previous ones and reinterprets observational and review studies at the molecular level. Our review provides evidence supporting the hypothesis of shared dysregulated molecular mechanisms between IR and PD, common to type 2 diabetes mellitus (T2DM) and PD individuals. PD is one of the most common motor and neurodegenerative disorders [9]. PD causes disability, and its prevalence is increasing worldwide [10]. This rise in PD cases has also been observed in Latin America [11] and highlights the importance of health policies to improve diagnosis, prevention, and treatment [12].

PD neurological features include the loss of dopaminergic neurons in the substantia nigra, and the presence of protein clumps called Lewy Bodies (LBs) in neurons. Movement disorders of PD include resting tremors, slowness, postural instability, and stiff neck [13]. Moreover, it has been reported that PD involves non-motor disorders like constipation, fatigue, insomnia, olfactory dysfunction, panic, lightheadedness, anxiety, and depressive symptoms [14,15,16], which could be detected several decades before motor disorders develop in PD individuals [17,18,19]. Early detection is a developing research field that could improve the diagnosis and treatment of PD [17].

Approximately 20% of PD cases have been associated with genetic inheritance, while the remaining cases are sporadic or idiopathic [13,20]. Genetic studies of PD have been performed using Next Generation Sequencing (NGS), which has allowed for the identification of several new loci that could explain PD development [21]. For instance, *SNCA* gene mutations are involved in LB production [22] and are the first genetic cause of PD in an autosomal dominant way [22,23,24,25]. These LBs are protein clumps of α-synuclein located in the cytoplasm of neurons [23]. Moreover, mutations in the *LRRK2*, *VSP35*, and *CHCHD2* genes have also been described as PD autosomal dominant causes, while mutations in *PRKN*, *PINK1*, *DJ*-1, *ATP13A2*, *FBXO7*, and *PLA2G6* genes as PD autosomal recessive [24,25]. Moreover, the role of the immune system in PD development is currently under research since it has been observed that there are shared gene mutations between PD and autoimmune disease [26,27].

IR has been associated with PD development and progression through mitochondrial dysfunction, reactive oxygen species (ROS) overproduction, and upregulated α-synuclein (*SNCA*) production [2]. Insulin is involved in the maintenance of brain homeostasis and its physiological functions. Hence, defective insulin signaling could contribute to PD development [5,28]. Also, α-synuclein aggregation in the nigrostriatal system has been observed in hyperglycemic mice models, suggesting an association between hyperglycemia and PD development [29].

As mentioned earlier, most PD cases have an idiopathic cause; however, a complex interaction between the genetic, immune system, age, and environmental factors has been suggested to contribute to developing PD clinical features [13]. For instance, it has been described that mutations in the major histocompatibility complex class II (MHC-II) locus, caused by insecticide exposure, could trigger a proinflammatory cell response and an increased PD susceptibility [30]. Similarly, heavy metals (lead, copper, and mercury) and illicit substances have also been associated as possible PD risk factors [20]. The molecular characterization involved in neuronal dysfunction after risk factor exposure could elucidate possible pathways implicated in neuronal pathogenesis and guide the development of new drugs for high-risk individuals.

Studies revealed that patients with IR and severe diabetes symptoms (chronic kidney disease, diabetic retinopathy, cardiovascular disease, hypoglycemic agents, and insulin use) have an increased PD risk [31]. Moreover, reports have associated Parkinson-like disorders with the overexpression of PED/PEA-15 in transgenic mice. PED/PEA-15 is a protein expressed in the brain and increased in T2DM patients [32]. Thus, the knowledge of the IR and T2DM molecular mechanisms involved in PD pathogenesis could contribute to the identification of new biomarkers for PD diagnosis.

Despite PD remaining uncurable, the treatment has been mainly focused on dopamine pharmacological substitution with levodopa (L-DOPA), an amino acid precursor, which reduces side effects and improves motor dysfunctions [32]. However, with long-term L-DOPA treatment, motor complications (dyskinesia, motor response oscillation) are observed [33]. These side effects boosted the development of several new dopaminergic drugs, which increases the half-life of L-DOPA and improves motor dysfunctions [33,34]. Moreover, several treatments have been developed in recent decades to improve PD symptoms. Some therapies modulate the neurotransmitters’ and neuromodulators’ non-dopaminergic signaling. These strategies include afferent, efferent, and intrinsic basal ganglia targets and pharmacological targets in the autonomic nervous system. These non-dopaminergic options focus on improving the non-motor and autonomic dysfunction in late-stage PD [33,34].

Furthermore, immunological treatment against α-synuclein protein aggregation, and anti-insulin resistance therapy, have been proposed to prevent neurodegenerative processes in PD patients by reducing α-synuclein aggregation [35]. Moreover, studies involving novel pharmacological approaches targeting insulin signaling have shown improvements in motor and cognitive dysfunctions; since these pathways are altered in PD and T2DM patients [36]. Hence, understanding the molecular mechanisms involved in IR or glucose metabolism dysfunction could allow new drug development to improve PD or type 2 diabetes mellitus outcomes.

Diet has also been linked with neurodegenerative symptoms, since malnutrition and insulin resistance have been described as risk factors and could trigger PD [37]. Moreover, the Mediterranean diet has been linked as a neuroprotective factor, due to the high consumption of fruits, vegetables, and fish, and low fat and refined carbohydrates intake [37,38]. Meat has been described as an external source of α-synuclein, which could act as prion-like particles and promote endogenous α-synuclein aggregation [38]. The regulation of oxidative stress is related to dietary macronutrient intake. The proportion of macronutrients ingested could deregulate the production of ROS and ATP in the mitochondria. Studies mention that protein intake increases oxidative stress and ROS levels, while carbohydrates produce less ROS than proteins and fatty acids [38]. Hence, a balanced diet rich in omega-3 fatty acids, antioxidants, and essential nutrients may have protective effects against both IR and PD.

**Figure 1:**
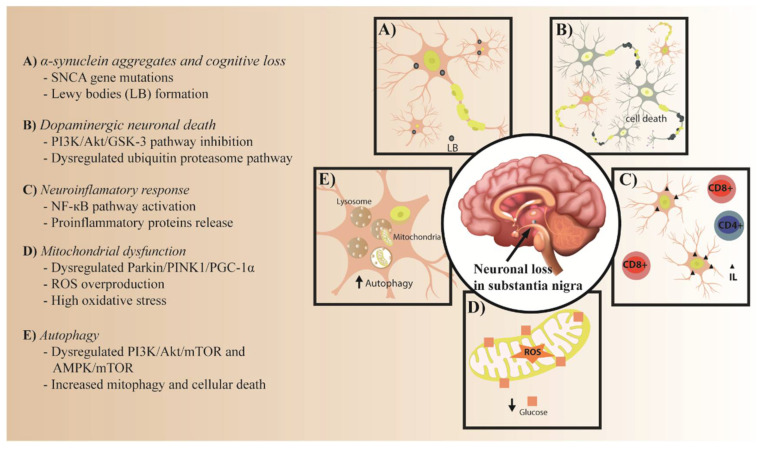
Common pathological process in PD and IR. (**A**) α-synuclein aggregates and cognitive loss. (**B**) Dopaminergic neuronal death. (**C**) Neuroinflammatory response. (**D**) Mitochondrial dysfunction. (**E**) Autophagy.

This review will describe the common molecular mechanisms involved in both IR and PD pathogenesis, and the role of IR as a possible risk factor for PD development. Lastly, possible therapeutic targets in insulin signaling for PD will also be discussed (Figure 1).

## 2. The Role of IR in PD Pathology

Insulin can be synthesized *de novo* in neurons and glial cells. Its primary function is to regulate glucose homeostasis in the whole organism, and its receptors are found in adipose tissue, skeletal muscle, liver, and other organs [39]. Furthermore, these receptors can also be found in the brain, explaining the role of insulin in brain activities such as cognitive function, appetite control, and homeostasis [6].

Dysregulation in insulin signaling and its side effects occur in patients with PD [39]. For instance, Bosco D. et al. 2012 mentioned that 60% of patients with PD and dementia presented IR [40], resulting in a decreased insulin action in specific tissues [41], which is a feature of the early stages of T2DM [31]. Hence, individuals with T2DM may have a higher risk of developing PD or worsening its symptoms [29,42].

Moreover, Kyungdo Han et al. 2023 evaluated the association between diabetes progression and PD risk. They found that, in a database of 2,362,072 T2DM patients with medical evaluations from 2009 to 2018, 0.72% (17,046) individuals were found to have PD. The severity of T2DM was determined by the characteristics presented by the patients, such as duration of diabetes, renal pathologies, and cardiovascular diseases. An analysis of these data led the authors to conclude that severe diabetes could be a risk factor for developing PD [31].

The relationship between elevated blood glucose and frequent dopaminergic neuronal loss in PD has been evaluated. In animal models overexpressing human α-synuclein protein and with streptozotocin (STZ)-induced T2DM, severe nigrostriatal degeneration and neuroinflammation were observed, suggesting that insulin resistance in T2DM affects the central nervous system [29].

Therefore, PD and IR share dysregulated biological processes and metabolic pathways, which include α-synuclein aggregation, dopaminergic neuronal loss, neuroinflammation, mitochondrial dysfunction, and autophagy [5] (Supplementary Table S1).

### 2.1. The Role of IR in α-Synuclein Aggregates

α-synuclein aggregation, a common feature of PD, may interfere with insulin signaling pathways, potentially promoting IR. Conversely, studies have shown that IR could also contribute to α-synuclein aggregation, neurodegeneration, and PD progression [2,43]. The α-synuclein is a small protein composed of 140 amino acids in length, encoded by the *SNCA* gene. This protein contains a positive N-terminal and an amphipathic region, which allows for the formation of an alpha-helical structure and the interaction with the lipids in the cell membrane. α-synuclein is highly present in the neurons, and possibly binding to synaptic vesicles of the pre-synaptic terminal [3,44]. Missense mutations in the N-terminal region of α-synuclein have been related to its dysfunction [3], which could promote the early onset of PD symptoms [45]. Furthermore, an *SNCA* locus triplication has been identified in PD individuals [46]. However, the specific cause for α-synuclein aggregation remains unclear. Several factors like environmental conditions, toxic substance interaction, gene expression levels, cellular membrane fatty acid concentration, and post-transcriptional modifications, among others, have been associated as triggering factors of α-synuclein aggregations [3].

Furthermore, α-synuclein monomers can aggregate into oligomers, which could form protofibrils that mature into fibrils with a β-sheet conformation that have been detected in LB of PD individuals [3,44,47]. The β-sheet-rich structure promotes α-synuclein aggregation, which could be caused by serine phosphorylation, ubiquitination, and C-terminal truncation [48,49]. Hence, oligomeric forms of α-synuclein have been associated with enhanced neurotoxicity [50] and abnormal α-synuclein aggregation, which could be a pathogenic factor in the development of PD [51].

Moreover, similar amyloid aggregates are observed in pancreatic β cells of DM2 patients [52], which could lead to the death and dysfunction of these types of cells [1]. Similarly, neuronal amyloid aggregates (α-synuclein) have been correlated with cell damage and death [53,54]. For instance, Hong, Chien-Tai et al. 2020 showed, in mice models, that IR-associated diabetes could promote PD progression via mitochondrial dysfunction, ROS overproduction, and enhanced SNCA signaling, leading to an increased α-synuclein production and subsequent aggregation [2].

Amyloidogenic pathway deregulation has been described in Alzheimer’s disease (AD) and PD. In this pathway, amyloid β precursor protein (type I transmembrane receptor-like protein) is cleaved by β-secretase and γ-secretase to produce amyloid β (Aβ) insoluble monomers. Then, these Aβ become Aβ plaques, which could alter insulin signaling and disrupt the PI3K/Akt pathway [55]. Moreover, this process could also occur in early PD stages, preventing insulin and IGF-1 from binding to their receptors (Ir, IGF-1r), leading to increased GSK3β activity by disrupting the PI3K/Akt pathway. The formation of LB and amyloid plaques trigger cognitive impairment (CI), a common PD characteristic [56]. Furthermore, CI has been associated with an increased risk of dementia in patients with T2DM and PD [57].

Likewise, it has been reported that polo-like kinase-2 (PLK2) is responsible for the phosphorylation of α-synuclein, potentially leading to α-synuclein aggregation [58]. Thus, the disruption of the PI3K/Akt pathway and the increased phosphorylation of α-synuclein by PLK2 promotes LB formation [59,60]. Therefore, PLK2 may be an important target for developing new pharmacological strategies.

### 2.2. PI3K/Akt/GSK-3 and Ubiquitin Proteasome Pathways in Dopaminergic Neuronal Death

Brain tissue consumes high amounts of oxygen and glucose to preserve cellular integrity [61]. The main component accountable for neuron integrity is the blood–brain barrier (BBB), which allows for the molecular exchange that maintains neuronal metabolism in the brain tissue [62]. Furthermore, insulin is responsible for glucose transport through BBB and ensures the energy supply for neurons [63]. In brain tissue, the PI3K/Akt/GSK-3 pathway and the Ubiquitin Proteasome Pathway are pivotal in regulating neuronal cell survival and apoptosis. IR promotes PI3K/Akt/GSK-3 pathway dysregulation, which has been implicated in neurodegenerative disorders, including PD [64,65].

#### 2.2.1. PI3K/Akt/GSK-3 Pathway

Dysregulated PI3K/Akt/GSK3β signaling is associated with neuronal oxidative stress and IR, which could promote cell apoptosis, α-synuclein aggregation, and early PD onset [66,67,68]. The PI3K/Akt/GSK3β signaling begins with the phosphorylation of insulin receptor (Ir) and insulin-like growth factor-1 receptor (IGF-1r) through the binding of insulin and insulin-like growth factor-1 (IGF-1), respectively. Phosphorylated Ir and IGF-1r activate insulin receptor substrate-1 and 2 (IRS-1/2), and subsequently the downstream phosphatidylinositol 3-kinase (PI3k) pathway. PI3k activates phosphatidylinositol 3, 4, 5-triphosphate (PIP3), which triggers phosphoinositide-dependent protein kinase (PDK), promoting protein kinase B (Akt) phosphorylation in threonine 308 and serine 473 residues. Phosphorylated Akt stimulates the phosphorylation of the serine 9 residue of the Glycogen synthase kinase β (GSK3β) to inactivate it. Phosphorylated GSK3β is a neuroprotective factor in the normal PI3K/Akt/GSK3β signaling pathway. However, when insulin and IGF-1 do not bind to their receptors (Ir, IGF-1r), it produces an under-phosphorylation of Ir and IGF-1r that triggers IRS-1 and IRS-2 hyperphosphorylation. Then, hyperphosphorylated IRS-1 and IRS-2 inhibit Akt, which activates GSK3β through dephosphorylation. Hyperphosphorylated IRS-1 and IRS-2 and activated GSK3β have been observed in the neurological loss of PD individuals [60]. Biochemical mechanisms have revealed that the IRS hyperphosphorylation process triggers PI3K inhibition. Neurotoxicity decreases by activating the PI3K/Akt metabolic pathway in normal conditions; however, these mechanisms related to Parkinson’s pathogenesis remain unclear and are still under investigation [60].

#### 2.2.2. Ubiquitin Proteasome Pathway

The ubiquitin-proteasome system (UPS) catalyzes the degradation of unfolded cytosolic proteins and allows for cellular homeostasis. Moreover, IR, by increasing the oxidative stress in the cell, could promote dysregulations in the UPS, which could lead to an α-synucleins aggregation, a hallmark of PD [69,70]. This enzymatic complex catalyzes the degradation of unfolded cytosolic proteins and allows for neuronal homeostasis. The UPS function begins with the ubiquitination in the N-terminal residues of the (misfolded) proteins. This process starts with the ubiquitin activation by binding to the E1 (ubiquitin activation enzymes). After that, activated ubiquitin joins the ubiquitin-conjugating enzymes (E2). Then, the activated ubiquitin-E2 conjugated is recognized by the ubiquitin ligase enzymes (E3), which transfer the ubiquitin to a protein substrate. At this point, E3 catalyzes the binding of several ubiquitin monomers at the first attached ubiquitin, promoting polyubiquitination. The complex polyubiquitin-protein is recognized by the proteasome, which lyses the protein (proteolysis) while ubiquitin is recycled. E3 dysfunction was reported to cause neuronal death in PD individuals. Dysregulated E3 promotes the proteolysis of neuronal pro-survival proteins, and the inhibition of the lysis of neuronal pro-apoptotic caspases [70,71]. Hence, the role of IR in the inhibition of UPS and its implication in PD development could be assessed. The mechanism of IR could be induced by the degradation of the insulin receptor, and other insulin signaling molecules via the UPS complex. In addition, proinflammatory molecules such as tumor necrosis factor-α, interleukins, and hypoxia-inducible factor 1α are regulated by ubiquitin E3, which regulates insulin signaling indirectly [72]. Another factor implicated in neuronal death is the depleted lysosomal function. Given that this organelle is implicated in the clearance of autophagosomes, a decline of lysosome function results in the accumulation of autophagosomes, which has been observed in the neurological tissues of post-mortem PD individuals [73]. Moreover, further research is needed to assess if IR is involved in lysosomal depletion through the inhibition of transcriptomic regulation of the structural proteins of the lysosomal membrane.

### 2.3. NF-κB Pathway in IR and the Neuroinflammatory Response

Chronic activation of the NF-κB pathway due to α-synuclein aggregates and inflammatory cytokines leads to neuronal inflammation in patients with PD. Moreover, this inflammatory process also impairs insulin signaling, disrupts glucose homeostasis, and promotes the development of IR [74]. PD neuroinflammation is associated with neuronal death, activated microglia, and peripheral immune cell aggregation [75,76], suggesting a relationship between the immune system and PD development [77,78]. Microglia play an important role in toxin protein removal, preventing neurodegeneration, activation against viruses or bacteria, and programmed neuronal cell death [77,79,80]. When microglia are activated, it releases several proinflammatory cytokines, interleukins, tumoral necrosis factor, pro-apoptotic proteins, reactive nitrogen, and reactive oxygen species, which are found in the neurons of the substantia nigra of postmortem PD patients [81,82]. These proinflammatory factors indicate a pivotal role of the innate immune system in the development of PD [83]. Moreover, the adaptive immune system is also involved in PD, since CD8^+^ and CD4^+^ T cell infiltration is found in the neurodegenerative tissue of postmortem PD individuals [83,84]. In conclusion, the microglia have an essential part in the maintenance of neurological homeostasis [13].

Neuronal death and proinflammatory microglia activation have been observed after treatment with 1-methyl-4-phenyl-1,2,3,6-tetrahydropyridine (MPTP) in dopaminergic neuronal cell culture and animal models. MPTP acts as an inhibitor of mitochondrial complex 1, and triggers ATP deficit and oxidative stress [85,86]. Hence, exposure to environmental toxins could lead to neuroinflammation and neuronal death.

Likewise, nuclear factor-κB (NF-κB) pathway is involved in the neuroinflammatory process. The gene expression of nitric oxide synthase, proinflammatory cytokines, and chemokines are regulated by transcription factors like NF-κB. NF-κB is activated after oxidative stress, which could be triggered by the inhibition of mitochondrial complexes I and III, by α-synuclein oligomers, or by the response to inflammatory molecules. NF-κB subunits are phosphorylated and then translocated from the cytosol to the nucleus. Then, NF-κB subunits bind to the κB site promoter and produce proinflammatory molecules that promote neuroinflammation [74].

The overactivity of the NF-κB pathway is a consequence of the increased binding of α-synuclein to immune receptors of the microglia [87]. These α-synuclein aggregates and LBs in neurons result from the insulin signaling disruption caused by high levels of the activated GSK3β kinase [60]. Hence, IR could promote neuroinflammation by overproducing proinflammatory cytokines inducing neuronal death and PD progression. GSK3β inhibitory molecules have been tested as PD therapeutic options [88]. However, due to GSK3β having an important physiological role in glycogen metabolism, its inhibition in neuroinflammatory processes must be fully evaluated.

### 2.4. Parkin/PINK1/PGC-1α Pathway in Mitochondrial Dysfunction

Mutations in the Parkin and PINK1 genes disrupt mitochondrial quality control and mitophagy processes, resulting in the accumulation of damaged mitochondria and increased oxidative stress in dopaminergic neurons. This cellular dysfunction is correlated to PD development. Additionally, IR has been associated with dysregulations in the Parkin/PINK1 pathway and contributes to mitochondrial metabolic dysfunction by reducing the activity of the PGC-1α coactivator [5]. Mitochondria generate most chemical energy (ATP) by oxidative phosphorylation; this energy is required for the normal functioning of all body tissues including heart, muscles, and brain [89]. Moreover, the brain is the organ with the highest energy consumption rate, either in an active or resting state [90]. In addition, the mitochondria are involved in processes such as Ca^2+^ ion homeostasis, which is necessary to activate signaling pathways and synapses [91]. The mechanisms of mitochondrial regulation, such as mitochondrial biogenesis, protein-complex formation, and mitochondrial protein post-translational processes, are dysregulated in individuals with IR and T2DM [92]. Furthermore, α-synuclein aggregates are related to the dysfunction of Ca^2+^ homeostasis, which triggers the impairment of the mitochondrial membrane. This mitochondrial Ca^2+^ stress leads to oxidative stress and cytochrome C release, which have been related to PD [93].

Studies suggest that in the skeletal muscle of insulin-resistant individuals, or those with T2DM, the mitochondrial density is 38% lower than in control individuals, which could trigger a decrease in mitochondrial function and dysregulation of metabolic pathways [94]. Moreover, cells affected by PD would have mitochondrial deregulation, which could promote the loss of dopaminergic neurons. The mechanisms that would be related to this pathology are genetic mutations, oxidative stress, environmental factors, mitochondrial DNA mutations, and epigenetic modifications [95].

Several mutations in the Parkin and PINK1 proteins are implicated in the development of familial PD [96]. Studies in animal models suggest that the Parkin/PINK1 pathway regulates mitochondrial quality control. Under normal conditions, Parkin and PINK1 proteins interact to eliminate nonfunctional mitochondria [97]. In addition, Parkin ubiquitinates the PARIS protein and promotes its proteasomal degradation. This degradation process allows for the activation of transcriptional peroxisome proliferator-activated receptor gamma coactivator 1 alpha (PGC-1α), which induces the gene expression involved in mitochondrial metabolism and biogenesis [96].

PGC-1α interacts with transcription factors such as NRF1 and NRF2 that regulate the transcription of mitochondrial respiratory chain proteins. Furthermore, the mitochondrial transcription factor (TFAM) is related to the oxidative stress response. Thus, dysregulations in PGC-1α could decrease the neuronal mRNA of transcription factors in neurons and be associated with PD and IR pathogenesis [98].

α-synuclein aggregates have been associated with mitochondrial integrity [99,100]. The high α-synuclein levels after insulin signaling dysregulation could trigger oxidative stress via mitochondrial fragmentation or mitochondrial membrane depolarization [101,102]. Therefore, oxidative stress contributes to the dysregulation of the Parkin/PINK1 pathway, related to mitochondrial dysfunction [98].

Thus, the loss of Parkin function triggers an accumulation of dysfunctional mitochondria, dysregulated mitophagy, oxidative stress, and dopaminergic neuronal loss in PD. Furthermore, Parkin dysfunction leads to the accumulation of PARIS and the inhibition of PGC-1α. The disruption of the Parkin/PINK1/PGC-1α pathway inhibits mitochondrial biogenesis and has been described in PD and IR individuals [103,104].

### 2.5. PI3K/AKT/mTOR and AMPK/mTOR Pathways in Autophagy

The PI3K/AKT/mTOR and AMPK/mTOR signaling pathways are regulators of autophagy. The hyperactivation of the PI3K/AKT/mTOR pathway, which has been observed in IR states, could inhibit autophagy, contribute to cellular dysfunction, and promote early PD onset. Conversely, a decrease in the AMPK/mTOR pathway activity due to IR could lead to an impaired autophagosome formation, inhibiting the degradation of unfolded proteins, and potentially promoting PD [42,105]. Autophagy and apoptosis are fundamental processes in the control of cellular homeostasis, and their dysregulation could be associated with some pathological conditions. In the case of insulin resistance and PD, autophagy processes have been correlated with the PI3K/AKT/mTOR metabolic pathway, which regulates synaptic survival and plasticity during aging and neurodegenerative diseases [67].

The PI3K/Akt metabolic pathway is activated by some IGF-1 and IGF-2 substrates, and the activated IGF-2 signaling is involved in neurite outgrowth and the regulation of autophagy. Furthermore, it was shown that the IGF-2 level was decreased in blood samples from patients with PD compared to healthy controls [106]. Additionally, the association of PI3K/Akt with the activation or inhibition of the mammalian target of rapamycin (mTOR) is another autophagy signaling pathway [107]. mTORC1 is an autophagy inhibitor, and mTORC1inhibition is associated with the elimination of misfolded proteins and dysfunctional organelles to avoid the accumulation of proteins related to the aging processes or neurodegenerative diseases [108,109]. Furthermore, some proteins involved in the autophagy process, such as LRRK2, PINK1, and Parkin, are present in mitophagy to destroy the impaired mitochondria [110].

Protein kinase AMPK regulates energy homeostasis through different metabolic pathways. The AMPK/mTOR metabolic pathway promotes autophagy through the activation of Unc-51-like autophagy activating kinase 1 (ULK1). ULK1 protein promotes phagosome formation by phosphorylating Beclin-1 to activate autophagy under starvation conditions [105,111]. In addition, AMPK is an antagonist of mTORC1 and inhibits the phosphorylation of target substrates affecting autophagy. The dysregulation of the AMPK pathway is involved with the development of neurodegeneration in diseases such as PD [112]. In patients with insulin resistance and T2DM, AMPK affects blood glucose and lipid metabolism. The alteration of energetic mechanisms can produce an altered synapse and cognitive impairment associated with PD [113]. Therefore, AMPK dysregulation promotes autophagy through ULK1 activation, the indirect mTORC1 inhibition pathway, and autophagosome and lysosome formation [105]. Furthermore, mitophagy is a defense mechanism for neurons, and studies have revealed that the activation of the AMPK/mTOR signaling pathway promotes: mitophagy, mitochondrial biogenesis, and the reduction of IR [112].

## 3. Potential Therapeutic Strategy for Insulin Resistance in PD

As previously described, PD is a chronic, neurodegenerative disorder with no cure [114,115]. The National Institute of Health recommends the use of levodopa for alleviating PD symptoms [116]. Levodopa (L-DOPA or L-3,4-dihydroxyphenylalanine) is a dopamine precursor, which, through the action of the aromatic amino acid decarboxylases within the glia and neurons, is converted into dopamine [117]. However, this type of treatment causes several side effects, including low blood pressure, nausea, vomiting, and neurotoxicity due to the formation of ROS [117]. PD patients usually take carbidopa to prevent the side effects of L-DOPA [116]. Therefore, it is vital to develop new alternatives to alleviate PD symptoms.

Drugs that target oxidative stress, inflammation, and insulin resistance, all of which lead to neurodegeneration, could also be a successful plan of action for PD [118]. For instance, exenatide, a manufactured version of exendin-4, can bind to the GLP-1 receptor and act as a peptide agonist, increasing the secretion of glucose-dependent insulin from pancreatic beta cells [118,119]. Research has shown that exenatide improves glycemic control in patients with T2DM and received FDA (Food and Drug Administration) approval in 2005 [119]. Furthermore, it has been demonstrated that exendin-4 at high doses can cross the BBB, induce neurite outgrowth, promote neuronal differentiation, reduce the loss of dopaminergic neurons, rescue degenerating cells, and may improve motor functions without causing cell toxicity [5,120,121,122,123,124]. Hence, the use of exenatide in PD treatment is currently being investigated.

Likewise, lixisenatide is another GLP-1 agonist based on the exendin-4 structure has received FDA approval for type 2 diabetes. Lixisenatide can cross the BBB, has neuroprotective properties, reduces motor function deterioration surpassing the effects of exenatide, and has anti-apoptotic properties [125]. Currently, there are Phase 2 clinical trials determining the use of lixisenatide for PD treatment. Liraglutide and semaglutide are two more examples of GLP-1 agonists studied as a possible treatment for PD [118,126].

Similarly, dipeptidyl peptidase-4 inhibitors (DPP4i) are used as hypoglycemic agents in type 2 diabetes treatment [127]. DPP4i has been correlated with improvements in glucose metabolism by inhibiting GLP-1 degradation, increasing its availability [128]. Furthermore, it has been demonstrated that DPP4i have neuroprotective properties, including anti-apoptotic signaling and the inhibition of neuroinflammation, and could decrease the risk of developing PD [7,129,130,131]. For instance, Badawi et al. 2017 showed that sitagliptin had a neuroprotective effect against nigrostriatal degeneration in rodents [8]. The DPP4i mechanisms of action are unclear regarding PD, since DPP4i cannot cross the BBB; it has been hypothesized that DPP4i could alleviate PD symptoms by increasing the blood levels of GLP-1 and GLP-2 [128].

Ghrelin has also been associated with the improvement of insulin signaling. Ghrelin is a hormone that is expressed in peripheral tissues and the central nervous system. Ghrelin has been reported to increase insulin sensitivity in neurons and decrease IR. Therefore, ghrelin could be a neuroprotective factor in neurodegenerative diseases such as PD [132,133]. Moreover, ghrelin can enhance several energy metabolic pathways like beta oxidation [133] and alleviate neuronal inflammation by reducing proinflammatory cytokines [134]. These characteristics open the possibility of studying the ghrelin pathway as a potential target in neurodegenerative diseases [132,133,134].

Additionally, increased vitamin E and omega-3 fatty acids in PD patients could improve the symptoms associated with PD, including the reduction of ROS production and dopaminergic cell death. Dietary restriction and exercise have also been shown to be beneficial for alleviating PD symptoms because of their effects on increasing dopamine levels and reducing glucose levels, and damage to neurons and associated motor dysfunction [36].

## 4. Discussion

Until now, PD has been one of the most prevalent neurological disorders with no cure. It is characterized by a reduced quality of life and a high cost of treatment [9]. In addition, the global burden of PD increases yearly due to aging and life expectancy improvement [135]. Thus, studies about infrastructure, medical care, and pharmacological and physical rehabilitation treatment costs could be performed to prevent the overburdening in neurological disorders management. For instance, a United States projection study estimates a PD prevalence of 1.6 million individuals produces more than USD 79 billion in economic burden [136].

By providing molecular analyses of the metabolic pathways common to IR and PD, we suggest possible pharmacological interventions that could ameliorate both. The evidence that incretin and insulin treatment improves insulin signaling and protects against neurodegenerative processes could be clinically tested as a neuroprotective application to subjects with PD [118]. Therefore, the evidence that insulin treatment can be neuroprotective in neuronal trauma [43] would support testing this approach against neurodegeneration and loss of insulin receptors and their function [137,138] in PD patients.

Aggregation and hyperphosphorylation of α-synuclein, which leads to LB formation, are key pathological features of PD, and both are associated with IR. IR promotes the expression of the SNCA gene which facilitates α-synuclein hyperphosphorylation and LB formation [2], contributing to PD motor and cognitive impairments [6]. So, targeting IR in PD could reduce insulin signaling disruption and ameliorate these key PD pathologies.

α-synuclein aggregation, a component of PD pathology, is linked to the UPS. Dysfunctional UPS and PI3K/Akt/GSK-3 pathways are associated with α-synuclein aggregation. Neurodegeneration in PD could be ameliorated by enhancing the E3 enzymatic complex of UPS or inhibiting deubiquitinase UPS14, an approach that is still currently under investigation [70]. Therefore, the specific characterization of the molecular mechanisms involved in PD pathogenesis through the impairment of insulin signaling, UPS, and the PI3K/Akt/GSK-3 pathway could help to develop new therapeutic options and improve the diagnosis.

Neurodegeneration in the form of reduced dopamine and other transmitter signaling in the hippocampus, cerebral cortex, and amygdala is a serious PD pathology, which is connected to IR in multiple ways [64]. It is triggered and enhanced by defective insulin signaling and improved with the correction of this defect. Evidence for the role of defective insulin signaling in PD pathology is fourfold. First, Glycatome, excessive hyperglycemia that results from IR, facilitates α-synuclein aggregation [29] and neuroinflammation that may promote PD development [139]. Glycatome reflects the protein glycation and disruption of several neuronal pathways in type 2 diabetes [139] and, thus, potentially in PD as well. Second, intestinal microbiota may mediate neuroinflammation in the brain, as the injection of α-synuclein aggregates into intestinal mucosa produces lesions in the neural enteric system [140,141,142,143], and the effect might be transmitted to the central nervous system through α-synuclein overproduction. Third, IR is associated with the malfunction of the PI3K/Akt pathway [144,145] and with impaired fatty acid metabolism, which may facilitate the emergence of α-synuclein [146], LBs [146,147,148], and neuronal death. Finally, a truncated insulin receptor promotes mitochondrial dysfunction, high ROS production, and the suppression of the PI3K/Akt pathway. The consequence is reduced dopamine signaling and the development of depression and anxiety in PD patients [149]. Other consequences are the reduced production of PGC1α and the associated depolarization of the mitochondrial membrane with the production of neurodegenerative ROS [2].

Neurodegeneration is prevented or mitigated by several molecular mechanisms that should be explored in clinical trials for the amelioration of PD symptoms. The first one is the neuroprotective action of IGF-1 by way of reducing α-synuclein aggregates [106,150]. IGF-1 concentrations may also mark the development of PD [150,151]. The second approach is to stimulate the insulin-degrading enzyme (IDE), which prevents α-synuclein and islet amyloid formation [152]. Conversely, the inhibition of IDE leads to the aggregation of α-synuclein in pancreatic α cells [153]. The third approach benefits from the similarities of PD and T2DM pathology of glucose intolerance. Increasing the glucagon-like peptide 1 (GLP-1) receptor decreases cognitive impairment in both metabolic disturbances and motor impairments in PD [154,155]. GLP-1 increases insulin secretion from pancreatic ß cells and suppresses glucagon secretion from this organ’s α cells [156]. The fourth approach derives from the effects of the GLP-1 receptor. It activates Akt signaling that regulates GSK-3B and mTOR proteins associated with inflammation, mitochondrial biogenesis, and autophagy [156]. Exenatide drug that activates GLP-1 receptors corrects deficiencies in dopaminergic and noradrenergic neurotransmitters in mice. Longer-acting agonists liraglutide and lixisenatide improve motor function and are neuroprotective [155]. The fifth approach to potentially ameliorate PD focuses on PPARγ (peroxisome proliferator-activated receptor), which reduces glucose levels and regulates the PARIS/PGC-1 α pathway. The activation of this pathway produces neuroprotective effects, modulates late inflammation, and enhances mitochondrial function [157]. Pioglitazone, a PPARγ agonist, inhibits dopaminergic neurodegeneration in rodents [158]. The inhibition of PARIS, and overexpression of PGC-1 α, could produce neuroprotective effects in PD patients [98].

The sixth potential therapeutic agent for PD is the enzyme protein kinase AMPK activated under the conditions of energy deficiency. Metformin activates the AMPK/mTOR pathway to improve PD motor symptoms [159], enhances autophagy, and delivers a neuroprotective effect [160]. Finally, exposure of PD subjects to a very low-calorie ketogenic diet suggests that this dietary approach may be neuroprotective and suppress neurodegeneration [161].

The limitations of the present review are mainly due to the heterogeneity of the results and the lack of conclusive studies; hence, more research is needed to clearly identify the association between insulin resistance and Parkinson’s disease.

## 5. Conclusions

PD is a neurodegenerative disorder that involves the interaction of environmental, genetic, immune, aging, and metabolic factors. Several neurological features like neuronal death, amyloid aggregation, neuroinflammation, autophagy, and mitochondrial dysfunction have been associated with both IR and different stages of PD development. Although the immune system has also been implicated in PD development; its association with IR remains under research. Currently, there is no cure for this pathology, and the available treatments are focused on attenuating PD symptoms that could generate side effects. Hence, the continuous research on the association between IR and the molecular and cellular mechanisms involved in neuropathogenesis is fundamental for the development of new pharmacological targets that improve PD outcomes and the identification of biomarkers that could help in PD diagnosis.

## Data Availability

The results are presented in the paper. For more information, please contact the corresponding author.

## Supplementary Materials

The following supporting information can be downloaded at: https://www.mdpi.com/article/10.3390/nu15163585/s1, Table S1: Pathways and the pathological processes involved in insulin resistance and Parkinson’s disease.

## Abbreviations

PD	Parkinson’s disease
IR	Insulin resistance
IGF-1	Insulin-like growth factor-1
AD	Alzheimer’s disease
LB	Lewy Bodies
NGS	Next Generation Sequencing
ROS	Reactive oxygen species
ENS	Enteric nervous system
IGF-1r	Insulin-like growth factor-1 receptor
IDE	Insulin-degrading enzyme
GSK3β	Glycogen synthase kinase β
GLP-1	Glucagon-like receptor
PLK2	Polo-like kinase-2
UPS	Ubiquitin proteasome system
PGC-1 α	Peroxisome proliferator-activated receptor gamma coactivator 1 alpha
mTOR	Mammalian target of rapamycin
PED/PEA-15	Phosphoprotein enriched in diabetes/phosphoprotein enriched in astrocytes-15
L-DOPA	Levodopa
STZ	Streptozotocin
Aβ	Amyloid β
CI	Cognitive impairment
BBB	Blood–brain barrier
IRS-1/2	Insulin receptor substrate-1 and 2
PI3k	Phosphatidylinositol 3-kinase
PIP3	3, 4, 5-triphosphate
PDK	Phosphoinositide-dependent protein kinase
Akt	Protein kinase B
MPTP	1-methyl-4-phenyl-1,2,3,6-tetrahydropyridine
T2DM	Type 2 diabetes mellitus
ULK1	Unc-51-like autophagy activating kinase 1
FDA	Food and Drug Administration
DPP4i	Dipeptidyl peptidase-4 inhibitors
