# Supplementary material for: The Molecular Mechanisms of the Relationship between Insulin Resistance and Parkinson’s Disease Pathogenesis
Source: Nutrients. 2023 Aug 15;15(16):3585. doi: 10.3390/nu15163585 (PMC10458139; doi:10.3390/nu15163585)
Supplement: Supplementary file 1 [file nutrients-15-03585-s001.zip › nutrients-2492896-supplementary.pdf]

Table S1. Pathways and the pathological processes involved in insulin resistance and Parkinson`s disease.

**Pathological process**	**Dysregulated Pathway**	**IR effects in PD**
α-synuclein aggregates	PI3K/Akt	Insulin signaling disrupted<br>α-synuclein overproduction<br>Abnormal α-synuclein folding<br>Lewy Bodies formation<br>Neurotoxicity
Dopaminergic Neuronal Death	PI3K/Akt/GSK-3<br><br><br><br><br>Ubiquitin Proteasome System	Ir and IGF-1r Under-phosphorylation<br>IRS-1 and IRS-2 hyperphosphorylation<br>Akt inhibition<br>GSK3β activation<br><br>Insulin receptor and insulin signaling molecules degradation<br>Ubiquitin ligase enzymes (E3) dysfunction<br>- Neuronal pro-survival protein proteolysis<br>- Neuronal pro-apoptotic caspase proteolysis inhibition
Neuroinflammation	NF-κB	PI3K/Akt/GSK3β pathway disruption<br>Overactivated GSK3β<br>- High α-synuclein levels<br>- NF-κB pathway upregulation<br>- Proinflammatory cytokines release
Mitochondrial dysfunction	Parkin/PINK1/PGC-1α	High α-synuclein levels<br>- Mitochondrial membrane depolarization<br>- Mitochondrial fragmentation<br>- Oxidative stress
Neuronal autophagy	PI3K/AKT/mTOR<br><br><br><br>AMPK/mTOR	Decreased IGF-2<br>mTOR inhibition<br>Misfolded proteins accumulation<br><br>Autophagy development<br>Energetic metabolism alteration<br>Synaptic and cognitive impairment
